# Sleep for cognitive enhancement

**DOI:** 10.3389/fnsys.2014.00046

**Published:** 2014-04-02

**Authors:** Susanne Diekelmann

**Affiliations:** Institute of Medical Psychology and Behavioral Neurobiology, University TübingenTübingen, Germany

**Keywords:** sleep, cognitive enhancement, memory, learning, reactivation, brain stimulation, pharmacology, ethics

## Abstract

Sleep is essential for effective cognitive functioning. Loosing even a few hours of sleep can have detrimental effects on a wide variety of cognitive processes such as attention, language, reasoning, decision making, learning and memory. While sleep is necessary to ensure normal healthy cognitive functioning, it can also enhance performance beyond the boundaries of the normal condition. This article discusses the enhancing potential of sleep, mainly focusing on the domain of learning and memory. Sleep is known to facilitate the consolidation of memories learned before sleep as well as the acquisition of new memories to be learned after sleep. According to a widely held model this beneficial effect of sleep relies on the neuronal reactivation of memories during sleep that is associated with sleep-specific brain oscillations (slow oscillations, spindles, ripples) as well as a characteristic neurotransmitter milieu. Recent research indicates that memory processing during sleep can be boosted by (i) cueing memory reactivation during sleep; (ii) stimulating sleep-specific brain oscillations; and (iii) targeting specific neurotransmitter systems pharmacologically. Olfactory and auditory cues can be used, for example, to increase reactivation of associated memories during post-learning sleep. Intensifying neocortical slow oscillations (the hallmark of slow wave sleep (SWS)) by electrical or auditory stimulation and modulating specific neurotransmitters such as noradrenaline and glutamate likewise facilitates memory processing during sleep. With this evidence in mind, this article concludes by discussing different methodological caveats and ethical issues that should be considered when thinking about using sleep for cognitive enhancement in everyday applications.

## Introduction

Sleep is vital to ensure normal human cognitive performance. Not obtaining enough sleep diminishes a wide variety of cognitive functions such as attention, language, reasoning, decision making, learning and memory (for reviews see Durmer and Dinges, 2005; Killgore, 2010; Jackson et al., 2013). Acute sleep deprivation of one or more nights without sleep can slow down reaction times (Van Dongen et al., 2003), increase perseveration (Retey et al., 2006), reduce focused attention (Thomas et al., 2000), impair risk assessment (Killgore et al., 2006) and strategic planning (Harrison and Horne, 1999), and deteriorate divergent thinking (Harrison and Horne, 2000) as well as language and communication skills (Harrison and Horne, 1998). Yet, does an impairment of cognition due to the loss of sleep in turn indicate that sleep *enhances* cognition? The answer is no. Evidence for deleterious effects of sleep loss demonstrates that sleep is necessary to enable normal healthy cognitive functioning. Cognitive enhancement, however, is defined as “the amplification or extension of core capacities of the mind” that go beyond the normal healthy condition (Sandberg, 2011). In this way, enhancement contrasts with therapy, which is understood as the treatment and prevention of diseases with the goal to restore or maintain the normal healthy condition (Bostrom and Roache, 2008). A number of methods have proven effective to enhance cognition in the state of wakefulness, such as pharmaceutical drugs, brain stimulation and meditation practices. Sleep is a state of the organism that differs fundamentally from the wake state with regard to the neurochemical milieu, brain activity patterns and mental states. This article reviews evidence for methods of cognitive enhancement during sleep. Sleep-specific manipulations have been found to effectively boost cognitive functions beyond the boundaries of the normal condition.

Cognition is not a unitary process but involves different functions and components. Sleep is effective in facilitating several of these functions such as language processing (Dumay and Gaskell, 2007), working memory (Kuriyama et al., 2008), creativity (Ritter et al., 2012) and decision making (Pace-Schott et al., 2012a). The largest amount of research on enhancing cognition during sleep, however, has focused on the domain of learning and memory (Rasch and Born, 2013). Memory is one of the most essential cognitive functions involved in almost all other cognitive processes. The ability to remember past experiences is not only critical for appropriate behavior in the present but it also allows us to anticipate and predict future events and situations. Our individual memories and our ability to learn new things are an integral part of our identity and influence our prospects of a successful and happy life. Considering that memory is of critical importance for individuals as well as for societies (e.g., in education, professional occupation, economic productivity, personal relationships), the possibility of enhancing memory has received increasing attention (Alberini and Chen, 2012; Collingridge et al., 2013; Stern and Alberini, 2013). Although sleep is widely neglected in the discussion of memory enhancement, the sleep state might be particularly well suited as a target for the enhancement of memory capacities.

Sleep is well known to facilitate memory consolidation, that is, the strengthening and stabilization of new memories acquired before sleep (Maquet, 2001; Walker and Stickgold, 2006; Rasch and Born, 2013). Subjects who are allowed to sleep after learning typically perform better on a subsequent retrieval test than subjects who spend a comparable amount of time awake following learning. Sleep actively promotes the reprocessing of fresh memories as well as their integration into the pre-existing network of long-term memories (Diekelmann and Born, 2010; Lewis and Durrant, 2011; Stickgold and Walker, 2013). Apart from its beneficial effect on the consolidation of previously learned memories, sleep also benefits the subsequent acquisition of new learning material (Van Der Werf et al., 2009). Even a short period of sleep prior to learning can enhance the capacity to encode new information (Mander et al., 2011).

Two main theories have been proposed to explain the beneficial effect of sleep for learning and memory. According to the active system consolidation theory, new memories become reactivated and reorganized on the system-level during sleep, with selected neuronal representations being potentiated and thereby strengthened (Lewis and Durrant, 2011; Inostroza et al., 2013; Rasch and Born, 2013). The synaptic homeostasis hypothesis, on the other hand, assumes that synaptic connections become widely depotentiated during sleep, whereby selected memory representations are depotentiated less or are spared from depotentiation so that these memories come out relatively stronger (Tononi and Cirelli, 2006, 2014). Both theories are well supported by experimental evidence and, importantly, are not mutually exclusive. Although this article is principally biased towards the active system consolidation theory, the reviewed evidence for cognitive enhancement during sleep is largely independent of mechanistic considerations and is also consistent with the synaptic homeostasis hypothesis in its most recent version (Tononi and Cirelli, 2014).

There are two ways in which sleep can be used to facilitate memory. First, sleep can be timed in relation to learning in such a way as to optimally support encoding and memory consolidation processes. In this way, the normal healthy function of sleep for memory can be optimized. For example, introducing short naps before learning of new information fosters the initial acquisition and encoding of new memory traces (Mander et al., 2011). Likewise, sleep episodes that follow shortly after the encoding session benefit the consolidation of fresh memories to preserve these memories for the long-term and protect them from subsequent interfering inputs (Gais et al., 2006b; Ellenbogen et al., 2009; Payne et al., 2012a,b). The second and more intriguing way to actually enhance memory during sleep (in the sense of augmenting memory beyond the normal condition) is to manipulate memory and/or sleep directly. Such manipulations target either the processing of specific memory representations during sleep or particular sleep features that are known to be implicated in memory processing. Additionally, pharmacological interventions can be applied to modulate the processing of memories during sleep.

The following sections Manipulation of Memory Reactivation During Sleep, Manipulation of Sleep-Specific Brain Oscillations and Manipulation of Neurotransmitter Systems outline examples of how memory representations and sleep processes can be directly or indirectly manipulated to enhance memory. Section Considerations and Caveats discusses possible considerations and caveats relating to methodological and ethical issues in the potential use of sleep for cognitive enhancement. Finally, section Conclusions presents general conclusions and an outlook on possible future research directions and practical applications.

## Manipulation of memory reactivation during sleep

The beneficial effect of sleep on memory is believed to rely on the neuronal reactivation of activity patterns that were present during the initial learning experience (Diekelmann and Born, 2010; Lewis and Durrant, 2011; Rasch and Born, 2013). In rats, firing sequences of hippocampal place cells that are observed during training, spontaneously re-express (“replay”) in a coordinated fashion during subsequent sleep periods (Wilson and McNaughton, 1994; Skaggs and McNaughton, 1996). Such replay mainly occurs during slow wave sleep (SWS; Lee and Wilson, 2002), sometimes at a faster firing rate than that of the original pattern (Nádasdy et al., 1999), and happens also in other brain regions such as the ventral striatum, visual cortex and prefrontal cortex (Euston et al., 2007; Ji and Wilson, 2007; Lansink et al., 2008). Reactivation of learning-associated brain activation patterns during sleep is also observed in humans, for example following training on a spatial navigation task (Peigneux et al., 2004) as well as a procedural serial reaction time task (Maquet et al., 2000). It is believed that these sleep reactivations strengthen the underlying memory traces and integrate them into long-term associative memory networks.

**Figure 1:**
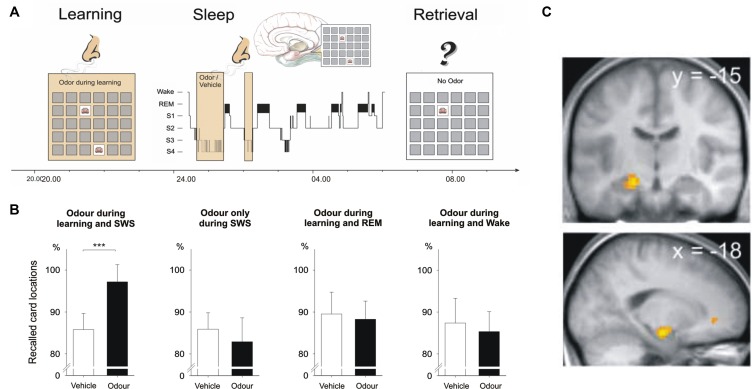
**Cueing reactivation during sleep by odors enhances memory. (A)** Subjects learned card pair locations in the presence of an odor. The same odor or an odor-less vehicle was then presented during subsequent slow wave sleep (SWS). **(B)** Recall of card pair locations in the next morning was significantly better when subjects had received the odor during SWS. Odor presentation during REM sleep and during wakefulness remained ineffective. Memory was also not enhanced when the odor was omitted during learning. **(C)** Presentation of the learning-associated odor during SWS activated hippocampal regions (activation in the left anterior hippocampus shown here). From Rasch et al. (2007). Reprinted with permission from the American Association for the Advancement of Science.

Intriguingly, memory reactivation does not only occur spontaneously during sleep but can be induced and/or intensified using externally applied reminder cues, a procedure that has been termed “targeted memory reactivation” (Oudiette and Paller, 2013). A ground-breaking study by Rasch et al. showed that memories for object locations can be reactivated during sleep by re-exposing subjects to odor cues that were previously associated with the learned object locations (Rasch et al., 2007). In this study, subjects learned to associate everyday objects and their locations in a 2D object location task, a task also commonly known as the game “concentration”. Throughout learning, subjects were presented with puffs of the scent of roses. Subjects went to bed after learning and during ensuing SWS the odor was presented again. When tested on their memory for the object locations in the next morning, subjects showed superior memory for the object locations when they had slept with the odor compared to another night in which they did not receive the odor during sleep (Figure 1). Odor re-exposure during rapid eye movement (REM) sleep as well as during wakefulness was not effective in enhancing memory performance. Also, presenting odor cues during SWS without prior odor exposure during learning had no effect on memory. Subsequent studies showed that odor-induced reactivation during SWS does not only strengthen memories but also transforms them into a more stable form making them resistant to subsequent interfering inputs (Diekelmann et al., 2011). Furthermore, memory reactivation triggered by external odor cues accelerates memory consolidation processes leading to stable memory representations after a short sleep interval of only 40 min—a process that takes roughly double that time without odor cueing (Diekelmann et al., 2012).

Triggering reactivation during sleep to enhance memory performance not only works with odor cues; auditory stimuli seem to be comparably effective. Although auditory memory cues have the disadvantage that they can potentially disrupt sleep patterns, they also provide an important advantage: while odors represent rather broad context cues that presumably reactivate the entire learning experience, tones and sounds are more specific and can be used to reactivate individual memory contents. Rudoy et al. presented pictures at specific locations that were paired with corresponding sounds (Rudoy et al., 2009). Subjects learned the location for each picture (e.g., cat) and listened to the associated sound (e.g., meow) during training. Half of the sounds were then presented again during a nap following the training session. After sleep, subjects were asked to place each picture in its original position. Subjects performed significantly better for those pictures whose associated sounds they had heard during sleep compared to the pictures for which sounds were not presented during sleep. Recent evidence suggests that similar memory enhancing effects can be obtained by cueing vocabulary during sleep. Schreiner et al. presented half of a previously learned set of Dutch-German vocabulary to their participants during post-learning SWS. In the next morning participants correctly remembered more of the words that had been cued during sleep compared to the vocabulary that had not been cued (Schreiner et al., submitted manuscript).

External reactivation during sleep can also enhance procedural memories of sequential finger tapping skills. In a study by Antony et al. subjects were trained to tap two different sequences repeatedly on a keyboard. The tapped sequences corresponded to a melody of specific tones presented simultaneously during sequence tapping training. During a post-training nap of about 90 min the melody of one of the sequences was presented again in SWS. When tested on both sequences after the nap participants showed better tapping performance in the sequence for which the corresponding melody had been played during sleep (Antony et al., 2012). Interestingly, this cueing effect is strongly specific for the individual tones presented during sleep. Cueing only parts of the trained melody sequence results in very specific enhancements of performance in the respective cued sequence finger transitions (Schönauer et al., 2014).

Triggering memory reactivation during sleep cannot only improve subsequent retrieval performance for desired memories but can also help to forget undesired memories. In a study by Hauner et al. human subjects underwent a contextual fear conditioning procedure in which pictures of faces were paired with mild electric shocks while specific odors were presented as contextual background. Presenting one of the contextual odor cues again during subsequent sleep reduced fear responses to the face images in the next morning (Hauner et al., 2013). A similar study in mice used an odor as conditioned stimulus that was systematically paired with foot shocks (Rolls et al., 2013). However, when mice were re-exposed to the odor during post-training sleep, subsequent fear responses to the odor were increased rather than decreased. Injection of a protein synthesis inhibitor in the amygdala before presentation of the odor during sleep resulted in fear extinction, i.e., reduced fear responses to the conditioned odor stimulus. These results demonstrate that subtle methodological differences in the experimental procedure can produce very different outcomes. For example, whether the reminder that is presented during sleep represents a context cue (as in Hauner et al.) or the conditioned stimulus itself (as in Rolls et al.) might determine whether fear memories are reduced or strengthened by reactivation during sleep (Oudiette et al., 2014).

Although the exact neurophysiological mechanisms underlying the effects of targeted memory reactivation during sleep are largely unknown, a seminal study by Bendor and Wilson indicates that external memory cueing during sleep can bias neuronal replay towards the firing patterns that were observed during prior learning (Bendor and Wilson, 2012). In this study, rats were trained on a linear track to run to the left side in response to sound L or to the right side in response to sound R. During subsequent sleep the sounds were presented again and sound L was found to elicit hippocampal firing patterns similar to those observed previously when the rats had been running to the left side during training, while sound R induced firing patterns similar to those observed when the rats had been running to the right side. Similarly, in humans the presentation of learning-associated cues during sleep activated task-specific brain regions in functional magnetic resonance imaging (fMRI). Re-exposure of a memory-associated odor during SWS activated hippocampal and neocortical brain regions (Rasch et al., 2007; Diekelmann et al., 2011), and presenting memory-related sounds during SWS increased activation in the parahippocampal cortex which predicted subsequent recall performance (van Dongen et al., 2012). Furthermore, epileptic patients who suffer from sclerosis in both hippocampi did not show a memory cueing effect with sounds during sleep, while in healthy controls as well as in patients with one functional hippocampus sound cueing enhanced memory consolidation (Fuentemilla et al., 2013). Thus, external memory reactivation biases neuronal replay and essentially depends on the integrity of the hippocampus. A recent study found that external memory cues (odors in this case) also alter sleep-specific brain oscillations presumably associated with memory replay. Odor cues presented during SWS increased slow wave activity (1–4 Hz) and fast spindle activity (13–15 Hz) and induced steeper slopes of slow oscillations, the latter being associated with memory improvement (Rihm et al., 2014).

## Manipulation of sleep-specific brain oscillations

Considering that specific field potential oscillations in the sleeping brain have been associated with memory consolidation during sleep, targeting these phenomena directly is another promising road to enhance memory. Several lines of research have implicated SWS as the sleep stage that is most prominently involved in memory consolidation (since the role of REM sleep and stage 2 sleep for memory is less clear, these sleep stages will not be considered here, but see for example Genzel et al., 2013; Ackermann and Rasch, 2014). Neocortical slow oscillations, thalamo-cortical spindles and hippocampal ripples are the hallmark oscillations of SWS that are associated with memory processing. Slow oscillations (<1 Hz) are characterized by global up-states (neuronal firing) and down-states (neuronal silence) and are believed to orchestrate the dialogue between hippocampus and neocortex for long-term memory storage (Sirota and Buzsaki, 2005). Specifically, spindles and ripples occur preferentially during the slow oscillation up-state (Steriade, 2006), which is a state of widespread neuronal activity. Spindles are characteristic waxing and waning oscillations (∼10–15 Hz) that seem to prime cortical networks for neuronal plasticity. Hippocampal ripples, on the other hand, are high-frequency oscillations of ∼100–300 Hz that accompany memory replay in the hippocampal networks and are believed to carry the actual “memory information”. The fine-tuned temporal interplay between slow oscillations, spindles and ripples presumably mediates the redistribution of new memories from the hippocampus, serving as a temporary store, to neocortical regions for longer-term storage (Diekelmann and Born, 2010).

**Figure 2:**
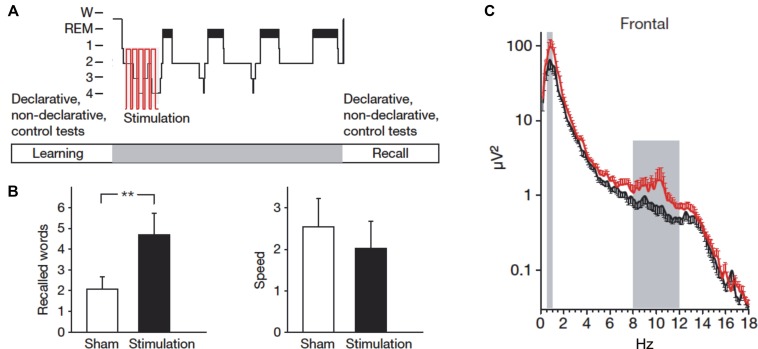
**Stimulating slow oscillations during sleep enhances memory. (A)** Following learning of word pairs and finger sequence tapping, subjects received electrical stimulation of slow oscillations (tDCS at 0.75 Hz) during ensuing sleep. **(B)** Compared to a sham condition, subjects remembered more word pairs after slow oscillation stimulation. Speed in finger sequence tapping was not affected by the stimulation. **(C)** The stimulation increased activity in the slow oscillation (0.5–1 Hz) and the slow spindle frequency band (8–12 Hz) at frontal sites. Reprinted by permission from Macmillan Publishers Ltd.: Marshall et al. (2006).

A seminal study by Marshall et al. found that intensifying slow oscillations by electrical transcranial direct current stimulation (tDCS) enhances memory consolidation (Marshall et al., 2006). Subjects studied a list of word pairs in the evening and during subsequent sleep an electrical current that oscillated at the peak frequency of the endogenous slow oscillations (i.e., ∼0.75 Hz) was applied to the subjects’ forehead. This electrical stimulation boosted endogenous slow oscillation activity and produced superior word recall in the next morning compared to a sleep condition without electrical stimulation (Figure 2). Importantly, the stimulation-induced memory improvement is specific for stimulation with the slow oscillation frequency: tDCS oscillating at 5 Hz (i.e., within the theta range) applied during SWS decreases slow oscillations and impairs memory consolidation (Marshall et al., 2011). Inducing slow oscillations can also be achieved with short auditory stimuli presented at the same frequency as endogenous slow oscillations (Ngo et al., 2013a). Presenting such an auditory stimulation closed-loop with endogenous slow oscillation up-states enhances memory consolidation of previously learned word pairs (Ngo et al., 2013b).

The stimulation of slow oscillations during sleep does not only enhance the consolidation of previously learned memories but can also augment the subsequent acquisition of new information. Antonenko et al. applied the previously described electrical slow oscillation stimulation during a midday nap in humans and tested their capacity to learn new word pairs, word lists and pictures thereafter (Antonenko et al., 2013). When subjects had slept with the slow oscillation stimulation, they were able to learn more new words and pictures after the nap compared to a nap without stimulation. Similar findings were observed in rats: electrical stimulation of slow oscillations via tDCS over several days improved the acquisition of task-inherent rules in a radial arm maze task (Binder et al., 2014). Intensifying slow oscillations might speed up processes that enable new learning in the hippocampus, possibly by down-scaling saturated neuronal networks or by shifting old memories from hippocampal to neocortical sites for long-term storage thereby freeing space for new memories.

Although spindles have frequently been associated with memory consolidation during sleep, it is unclear whether sleep spindles can be externally manipulated. There are numerous studies showing that spindle activity as well as spindle density increase following learning (e.g., Gais et al., 2002; Schabus et al., 2004; Schmidt et al., 2006) and the number and activity of sleep spindles correlates with subsequent memory performance (e.g., Clemens et al., 2005; Nishida and Walker, 2007). Furthermore, reactivating memories with learning-associated odors enhances spindle activity during odor cue presentation in SWS (Rihm et al., 2014). Increasing slow oscillations by electrical and auditory stimulation likewise induces a concurrent increase in spindle activity (Marshall et al., 2006; Ngo et al., 2013b). Yet, whether the relationship between spindles and memory is a causal one is unclear. It remains to be elucidated whether spindles can be externally triggered and whether such triggered spindles prove effective in enhancing memory.

Like sleep spindles, hippocampal ripple events are commonly associated with memory consolidation during sleep. During SWS in rats, ripples are observed during the replay of hippocampal neuron ensembles (Wilson and McNaughton, 1994; Peyrache et al., 2009) and their occurrence increases following learning (Eschenko et al., 2008). Moreover, during a nap in humans the number of ripples correlated with the consolidation of previously learned picture memories (Axmacher et al., 2008). Suppressing hippocampal ripples by electrical stimulation during post-learning sleep impairs memory for previously learned tasks in rats (Girardeau et al., 2009; Ego-Stengel and Wilson, 2010). However, it is hitherto unknown whether ripples can be induced or increased by external stimulation and whether this would enhance memory consolidation and/or acquisition.

## Manipulation of neurotransmitter systems

A number of different neurotransmitters and hormones modulates and influences the formation of memories. Most of these neurotransmitters and hormones are differentially regulated during sleep and wakefulness. SWS in particular is characterized by a decrease in levels of acetylcholine, noradrenaline and cortisol as well as by a distinct increase in growth hormone concentrations compared to periods of wakefulness (Marrosu et al., 1995; Born and Fehm, 2000). Other neurotransmitters such as glutamate, dopamine and GABA exhibit changes in concentrations across the sleep-wake cycle as well (Sowers and Vlachakis, 1984; Dash et al., 2009; Vanini et al., 2012). The exact mechanisms underlying the specific role of neurotransmitters in memory processing during sleep are not well understood. It is believed that the different levels of the respective neurotransmitters allow for and mediate the replay of firing patterns in neuronal circuits as well as the associated electrophysiological brain oscillations during sleep.

Several neurotransmitters have been found suitable for memory enhancement during sleep. For example, increasing the availability of noradrenaline during sleep with the noradrenaline reuptake inhibitor reboxetine enhances performance in an odor recognition task (Gais et al., 2011). In this study, subjects learned a set of odors and received reboxetine or placebo before a night of sleep. Two days later subjects recognized more of the previously learned odors when they had received reboxetine as compared to the night with placebo administration. In another study, reboxetine enhanced the consolidation of procedural memories in a finger sequence tapping task, which was associated with an increase in the number of sleep spindles (Rasch et al., 2009). The role of noradrenaline in sleep-dependent memory consolidation is further supported by findings showing that reducing the availability of noradrenaline by administering the α2-autoreceptor agonist clonidine blocks the improvement of memory for odors (Gais et al., 2011) as well as the enhancement of emotional over neutral memories that is typically observed after sleep (Groch et al., 2011).

Enhancing glutamatergic neurotransmission during sleep likewise boosts the consolidation of previously acquired memories. Feld et al. performed a study in which subjects learned a set of word pairs in the evening and received the NMDA receptor coagonist D-cycloserine during subsequent sleep (Feld et al., 2013a). At retrieval testing 1 day later subjects remembered significantly more of the learned words when they had received D-cycloserine during sleep compared to a night of sleep without substance administration. D-cycloserine is assumed to facilitate synaptic plastic changes resulting from reactivation of glutamatergic neuronal ensembles (Feld et al., 2013a).

Other substances that target different neurotransmitter systems can enhance the consolidation of particular memories during sleep. For instance, the dopamine D2-like receptor agonist pramipexole enhances the consolidation of memories that had been associated with low reward during learning before sleep but does not affect memories associated with a high reward (Feld et al., in press). The cytokine interleukin-6, administered intranasally shortly before sleep, increases slow wave activity and enhances the consolidation of emotional but not neutral memories (Benedict et al., 2009). And while reboxetine increases the consolidation of finger sequence tapping skills it does not affect mirror tracing performance (Rasch et al., 2009).

Studies on the effect of GABA for memory enhancement during sleep revealed mixed results (Hall-Porter et al., 2014). Increasing spindle density with the GABA_A_ positive modulator zolpidem enhances memory for word pairs and emotionally negative pictures but impairs perceptual learning and has no effect on finger sequence tapping and memory for emotionally neutral pictures (Kaestner et al., 2013; Mednick et al., 2013). Intensifying SWS and slow wave activity with the GABA reuptake inhibitor tiagabine did not have an enhancing effect on memory consolidation (Feld et al., 2013b), which was possibly due to a non-functional increase of slow oscillations that was accompanied by a decrease in spindle activity phase-locked to slow oscillations. Growth hormone, another important neuromodulator, had long been suspected to play a role in sleep-dependent memory consolidation as it is mainly secreted during SWS and is involved in hippocampal memory formation (Nyberg and Hallberg, 2013). Yet, effectively blocking growth hormone release during SWS by infusion of somatostatin left memory consolidation unaffected (Gais et al., 2006a). Whether it is possible to enhance memory by increasing the availability of growth hormone during sleep is unclear.

Low levels of the neurotransmitter acetylcholine are known to be critical for SWS-dependent memory consolidation (Buzsáki, 1986; Hasselmo, 1999). Increasing cholinergic activity during SWS by administering the choline esterase inhibitor physostigmine impairs memory consolidation (Gais and Born, 2004). It is unclear, however, whether a reduction of cholinergic activity during SWS can further boost memory. Findings of studies that manipulated the stress hormone cortisol during SWS suggest otherwise. Cortisol shows secretion patterns very similar to those of acetylcholine, with very low concentration levels during SWS. Similar to acetylcholine, increasing cortisol during SWS by infusion of hydrocortisone or dexamethasone blocks the beneficial effect of sleep for memory consolidation (Plihal and Born, 1999; Plihal et al., 1999; Wilhelm et al., 2011). Yet, further decreasing cortisol levels during SWS with the cortisol synthesis inhibitor metyrapone did not enhance but paradoxically reduced the consolidation of hippocampus-dependent memories (Wagner et al., 2005). Thus, the neurochemical milieu of neurotransmitters and hormones during sleep, particularly during SWS, might be intricately optimized to support memory consolidation such that pharmacological manipulations of this finely tuned balance often do not have the expected results.

Overall, manipulating neurotransmitter systems to enhance memory during sleep has revealed inconsistent results. Future research will have to replicate and specify the key findings in this field. At present, these findings should therefore be interpreted with caution.

## Considerations and caveats

Several different manipulations of sleep and memory have been proven effective to enhance learning and memory consolidation. Yet, there are a number of central questions that are still unknown, both in the experimental study of sleep to enhance memory as well as for possible applications of these new methods in everyday life. Among these questions are important issues relating to methodological intricacies, such as possible boundary conditions, potential unintended effects, the actual effect size of the improvement, as well as the practicability for everyday use. Besides these methodological problems, the possibility of enhancing cognitive functions during sleep raises certain ethical questions. Apart from the ethical questions that are discussed in relation to cognitive enhancement in general, applying cognition-altering manipulations during sleep—a state of unconsciousness, reduced voluntary control, and heightened vulnerability of the individual—might pose additional ethical concerns.

### Methodology

Although we know that normal sleep facilitates learning and memory and that specific manipulations of memory during sleep can further boost this effect, we still don’t understand the exact conditions under which the memory enhancement occurs (Diekelmann et al., 2009; Diekelmann, 2013). Memory cueing, stimulation of sleep oscillations as well as pharmacological interventions enhance some memories but not others depending on the particular methods used. For example, using odors as context cues to reactivate associated memories during sleep enhances declarative memories for object locations but not procedural memories in the finger sequence tapping task (Rasch et al., 2007). Stimulating slow oscillations via tDCS likewise enhances declarative word pair memories but not procedural finger sequence tapping (Marshall et al., 2006). Yet, cueing memories for finger sequence tapping directly by tones associated with specific finger movements does in fact enhance finger tapping performance (Antony et al., 2012; Schönauer et al., 2014).

Furthermore, increasing the availability of noradrenaline during sleep by reboxetine enhances odor memory (Gais et al., 2011) and finger sequence tapping (Rasch et al., 2009) but does not enhance memory for word pairs and performance in a mirror tracing task (Rasch et al., 2009). Intranasal interleukin-6 improves memory for emotional stories but not for neutral stories, object locations and finger sequence tapping (Benedict et al., 2009). And while the modulation of GABA transmission with zolpidem enhances emotional picture memory and verbal memory, it does not affect neutral picture memory and motor memory, and it even impairs perceptual learning.

Considering that some memories can be enhanced during sleep under some conditions but not under others suggests that there are certain boundary conditions for the enhancing effect of sleep. For cueing memory reactivation with odors, for example, it was found that the same odor needs to be presented during learning and subsequent sleep to enhance memory; presenting a different odor during sleep than during learning remains ineffective (Rihm et al., 2014). Generally, it is unclear how many different odors can be associated with different memories and how many times a single odor can be associated with new learning material before it produces interference rather than enhancement. Olfactory cues presumably represent general context cues that reactivate the entire learning context when presented during sleep, increasing the risk of interference when different memories are learned in the same context. In order to target individual memories, auditory cues might be better suited; yet these cues have the disadvantage to change the natural sleep cycle by inducing arousals and awakenings if the sound level is not optimally adjusted. The effectiveness of memory cueing during sleep might generally depend on whether or not the learning information is relevant for the individual, which can be signaled for example by associated low or high rewards (Oudiette et al., 2013). Furthermore, when trying to reduce undesired memories by cueing during sleep, different methods can produce fundamentally different results. Whether fear memories are weakened or even strengthened during sleep, for instance, depends on methodological subtleties such as contingencies in the conditioning procedure and the nature of the reactivation cue (Oudiette et al., 2014).

Similar boundary conditions are presumably relevant in the stimulation of sleep-specific brain oscillations as well as in pharmacological manipulations of neurotransmitters and hormones. Importantly, sleep oscillations, neurotransmitter concentrations and memory replay during sleep are intricately interlinked. Targeting one or the other component of this interwoven net of factors might produce unexpected adverse effects and can even be harmful to memory. For example, the GABA agonist tiagabine increases slow wave activity but does not enhance memory consolidation, possibly due to a concurrent decrease in functionally coupled spindle activity (Feld et al., 2013b). Likewise, low levels of cortisol during SWS are known to be essential for memory consolidation processes, yet further decreasing cortisol concentrations paradoxically impairs memory rather than further enhancing it (Wagner et al., 2005). These examples show that it is essential to consider the functional role of single components and processes in sleep-dependent memory consolidation as well as their inter-dependencies when trying to manipulate particular aspects of this complex system. However, the way in which the different processes and components interact is not well understood.

Apart from its enhancing effect on memory, manipulations of memory processing during sleep can have side effects and unintended effects. It has been shown, for instance, that the reprocessing and integration of information during sleep can qualitatively change memories. Although in many cases this a positive effect, for example generating insight (Wagner et al., 2004), drawing relational inferences (Ellenbogen et al., 2007) and abstracting schemas (Lewis and Durrant, 2011), it can also lead to the generation of memory distortions and false memories (Payne et al., 2009; Diekelmann et al., 2010). Up to now it is unclear whether and how external manipulations of memory during sleep affect the veridicality of memories. Furthermore, there might be a trade-off between different memories: enhancing one memory could come at the cost of impairing others. For example, sleep increases memory for emotional components of a scene but concurrently decreases memory for the neutral background (Payne et al., 2008, 2012a; Payne and Kensinger, 2010, 2011). Although trade-off effects in sleep-dependent memory consolidation have not been studied directly, indirect evidence suggests that there might be a limited capacity for memory replay during sleep such that increasing reactivation of one memory decreases reactivation of other memories (Antony et al., 2012; Bendor and Wilson, 2012; Kelemen and Born, 2012). It is generally unknown how targeted manipulations of memory during sleep are. The discussed manipulations might not only affect other memories but also other cognitive functions and even other bodily functions. Slow oscillation stimulation with tDCS, for example, was found to improve mood, though the mechanisms for this effect are unclear (Marshall et al., 2006; Göder et al., 2013). Also pharmacological treatments for memory enhancement during sleep are typically “dirty” interventions that affect numerous other functions and can also produce unwanted side effects.

Although the effects of memory enhancement during sleep are reproducible and statistically significant, the practical significance of these effects is unclear. In humans, the estimated performance improvement in studies manipulating memory during sleep (either by cueing memory replay, stimulating sleep oscillations or applying pharmacological substances) is usually in the margins of 5–15% compared to sleep conditions without treatment. Considering that natural sleep without any manipulations produces a memory improvement of about 10–20% compared to wakefulness, the combined improvement of sleep with external manipulations adds up to an enhancement of 15–35% as compared to wake intervals without manipulations. Even taking into account that this number might eventually turn out to be smaller, considering that effect sizes are typically overestimated in the first studies of a new field of research, the true effect size might still be high enough to warrant practical applications. Experimental studies on sleep and memory usually test for enhancing effects of sleep manipulations during one night of sleep only. The long-term effects of using sleep applications for cognitive enhancement on a regular basis are presently unclear. Generally, the effect of sleep and the need for sleep is rather variable between individuals. Considering that current activity schedules, in schools and at work, do not provide an ideal environment for adequate sleep, the most straight forward method to optimize normal cognitive function by sleep in a first step may be a policy change towards more flexible timing of activity and rest.

The practicability of memory enhancing methods during sleep for applications in everyday life depends on the type of method. Odors are relatively easy to apply during learning and subsequent sleep in the home environment. For auditory cues, home applications are likewise relatively easy to conceive. Stimulation of sleep-specific brain oscillations and pharmacological substance administration, however, is more difficult to do in everyday life. Although apparatuses for home use of tDCS in the wake state are already available on the market, their potential for the stimulation of specific sleep oscillations is unclear. Also safety and potential side effects of long-term tDCS treatment is completely unknown. The optimal stimulation of sleep-specific brain oscillations as well as the application of odors and sounds to reactivate memories during specific sleep stages is further complicated by the lack of easy-to-use sleep recording systems for the home environment. Finally, the pharmacological substances that (so far) have been shown to enhance memory during sleep are not available for use outside their medical indication at the moment.

### Ethical issues

On the one hand, ethical questions regarding the use of sleep for cognitive enhancement relate to the general ethical issues that are discussed in the context of cognitive enhancement and biomedical enhancement at large (for overview see for example Farah et al., 2004; Buchanan, 2011). The enhancement of cognitive capacities can potentially have substantial positive effects for individuals as well as for societies by increasing productivity and overall quality of life through more personally and financially rewarding occupations, more fulfilling personal relationships, and less suffering from cognitively impairing diseases (Buchanan, 2008, 2011; Bostrom and Roache, 2011). Yet, cognitive enhancement might also have direct or indirect negative consequences. Just to name a few, through the enhancement of our cognitive capabilities we could lose our sense of giftedness (Sandel, 2007), we might no longer appreciate the effort to achieve something, we could become inauthentic by changing central features of our identity such as our memories, we might be coerced to use cognitive enhancers if others do it, the use of cognitive enhancers might be seen as cheating, and an unfair access to cognitive enhancers could potentially increase social injustice.

Ethical issues that are specific to manipulations of cognitive capacities by targeting sleep relate to the specific nature of sleep as an exceptional state of the organism. Sleep is characterized by immobility and muscle relaxation, greatly diminished processing and perception of external stimuli as well as greatly limited or even absent voluntary control. These characteristics make the sleeping individual particularly vulnerable for all kinds of external influences. Manipulations of cognitive processes during sleep are designed in such a way that the individual does not notice the treatment. In studies using memory cueing during sleep, subjects typically report not to have noticed any presentation of the cues. Likewise, following stimulation of brain oscillations during sleep as well as after the administration of pharmacological substances, participants usually cannot tell whether they received the treatment or not. Thus, subjects not only have no control over such manipulations but they don’t even know whether they have been manipulated at all. This constellation can give rise to a certain misuse potential. In Aldous Huxley’s science fiction novel “Brave new world”, indoctrination of infants and children during sleep is used to shape how these children will think, feel and behave as members of their respective caste. Huxley calls this procedure “hypnopaedia” and describes it as “the greatest moralizing and socializing force of all time”. Although it is unclear whether sleep goes along with an enhanced suggestibility, Huxley’s fictitious idea illustrates that sleep is a highly sensitive state in which we are relatively unprotected against external influences.

Such worries and premonitions are legitimate and important to discuss in the context of cognitive enhancement during sleep. However, they do not provide conclusive reasons against the use of sleep for cognitive enhancement. What is needed is a balanced account to weigh possible risks against the benefits to allow for a well-informed and all-things-considered judgment about the responsible use of sleep for cognitive enhancement (Buchanan, 2011). Even if negative consequences were to be expected, the numerous positive effects could be so desirable as to outweigh possible negative effects. The question is not whether or not to use sleep for cognitive enhancement—cognitive enhancers of all sorts are already there and their use will increase even more in the near future—the question is rather how to enhance cognitive capacities responsibly, how to deal with issues of distributive justice and how to prevent misuse and malpractice.

## Conclusion

This article has focused on the function of sleep to augment cognitive capacities, a function that will arguably receive more and more attention in the next few years, both in research as well as in practical applications. The reviewed evidence shows that targeted manipulations of memory processing during sleep can be used to enhance learning and memory. Sleep can thereby be considered a cognitive enhancer as this state provides optimal conditions to augment memory capacities above and beyond the normal level. It is important to note that the discussed effects of manipulations to enhance memory during sleep are specific for the sleep state. Identical methods applied during wakefulness have no enhancing effect on memory or can even impair memory processing. For instance, reactivating memories by odors during wakefulness does not affect memory for object locations (Rasch et al., 2007) or even renders these memory representations unstable making them more vulnerable for disruptive interfering inputs (Diekelmann et al., 2011). Manipulating sleep-specific brain oscillations is naturally confined to the sleep state. Except for ripples, which also occur during wakefulness (O’Neill et al., 2010), slow oscillations and sleep spindles selectively occur during sleep. Interestingly, stimulating the brain during wakefulness with frequencies oscillating in the slow oscillation range has no effect on memory consolidation but increases theta activity resulting in better encoding of new information (Kirov et al., 2009). Furthermore, the neurochemical milieu of neurotransmitters and hormones is very different during sleep and wakefulness such that administration of certain substances can have fundamentally different effects on memory. For example, while an infusion of cortisol impairs memory consolidation during sleep it facilitates consolidation processes when administered in the wake state (Wilhelm et al., 2011).

Importantly, memory enhancement in the context of cognitive enhancement should not be confused with the term “enhancement” that has sometimes been used in the sleep and memory literature to describe actual sleep-dependent gains in performance. Initial research on memory consolidation during sleep, particularly with respect to procedural memory, showed that performance levels after sleep can exceed performance levels observed before sleep (Walker, 2005). We know now that this is not true (see for example Rickard et al., 2008). Even external manipulations of memory during sleep cannot produce anything that was not previously encoded; rather sleep as well as memory manipulations during sleep enhance memory in the sense of less forgetting (to the point of no forgetting) and higher memory stability (i.e., resistance to subsequent interference). Related to this, only a very few reports show that conditioned responses can be acquired during sleep (Arzi et al., 2012), but new learning of declarative and procedural memories during sleep seems to be impossible. Nevertheless, sleep can produce new knowledge from previously encoded memories through processes of generalization, integration, schema abstraction, and conversion of implicit into explicit knowledge (Wagner et al., 2004; Gomez et al., 2006; Ellenbogen et al., 2007; Durrant et al., 2011; Wilhelm et al., 2013).

It is of note that sleep is not only effective as a cognitive enhancer to boost memory performance, but it can also be applied in clinical settings to restore normal cognitive functioning. A number of disorders and diseases are accompanied by changes in sleep patterns and dysfunctions of memory, such as depression (Steiger et al., 2013), post-traumatic stress disorder (Germain, 2013), Alzheimer’s disease (Wang et al., 2011) and schizophrenia (Lu and Goder, 2012). In patients with schizophrenia, stimulation of slow oscillations with tDCS during sleep was found to improve memory for words (Göder et al., 2013). Furthermore, administration of the atypical antipsychotic olanzapine increased SWS, and the GABA agonist eszopiclone enhanced the number of sleep spindles in schizophrenia; yet both treatments failed to normalize memory consolidation in these patients (Göder et al., 2008; Wamsley et al., 2013). Future studies will have to test new methods for the treatment of memory dysfunctions during sleep in different clinical settings, which could also potentially improve the outcome of standard therapeutic treatments. For the treatment of spider phobia, for example, sleep after exposure therapy was found to increase therapeutic effectiveness (Pace-Schott et al., 2012b; Kleim et al., 2013). It remains to be elucidated whether manipulations of memory processing during sleep, such as memory reactivation or sleep oscillation stimulation, can boost this effect further.

Manipulating memory during sleep is a very new field of research and we are only beginning to understand which methods are best suited to use sleep for memory enhancement. Much more research is needed to determine the optimal procedures to unravel this effect and to understand its underlying mechanisms and principles. Future research will have to extend the knowledge obtained from findings in the domain of learning and memory to other cognitive functions such as creativity, executive functions and decision making. This research could not only provide us with new means to augment our cognitive capabilities in different domains but could also help to change the image of sleep as a useless waste of time to being a beneficial state that supports our wake performance.
